# The nexus between geographical distance and institutional delivery trends in Ethiopia: evidence from nationwide surveys

**DOI:** 10.7717/peerj.18128

**Published:** 2024-09-20

**Authors:** Yemisrach Berhanu Sebsibe, Tayue Tateke Kebede

**Affiliations:** 1 School of Public Health and Community Medicine, University of Gothenburg, Gothenburg, Sweden; 2 Faculty of Health Sciences, Kristianstad University, Kristianstad, Sweden; 3 Institute of Medicine, University of Gothenburg, Gothenburg, Sweden

**Keywords:** Accessibility of health services, Facility delivery, Institutional delivery, Distance to health facilities, Maternal health, Facility distance

## Abstract

**Background:**

Giving birth in a healthcare facility with the guidance of skilled healthcare providers allows access to necessary medical interventions. Ethiopia has implemented several strategies to enhance institutional delivery and decrease maternal mortality; however, the rate of institutional delivery remains low. This study examines the role of distance to healthcare institutions on institutional delivery in Ethiopia, and how this has changed over time.

**Method:**

This study used data from two rounds of the Ethiopian Demographic and Health Survey (2011 and 2016), a spatial database detailing the locations of healthcare facilities, and Ethiopian road network data. The sample included 22,881 women who delivered within the 5 years preceding each survey and lived in 1,295 villages. Bivariate and multivariable logistic regression analyses were used to investigate how the distance to health facilities and other potential determinants influenced institutional delivery trends.

**Results:**

The rate of institutional deliveries in Ethiopia has increased from 10% in 2011 to 26% in 2016. Likewise, the average transportation distance to health facilities has decreased from 22.4 km in 2011 to 20.2 km in 2016 at the national level. Furthermore, a one-kilometer increase in the distance to the nearest health facility was associated with a 1% decrease in the likelihood of delivering at a health facility in 2016 (odds ratio (OR) = 0.99, 95% CI [0.98–0.99], *p* < 0.05). Additionally, mothers who are more educated, have completed more antenatal care visits, live in wealthier households in more urban areas, and cohabit with more educated husbands are more likely to deliver at healthcare facilities. These variables showed consistent relevance in both survey rounds, suggesting that key determinants remained largely unchanged throughout the study period.

**Conclusion:**

The impact of distance from health facilities on institutional delivery in Ethiopia remains evident, although its influence is relatively modest. The other factors, including education, antenatal care, socioeconomic status, urban residence, and partner education, remained consistent between the two surveys. These determinants have consistently influenced institutional delivery, highlighting the importance of a comprehensive approach that addresses both access to and socioeconomic factors to improve maternal and infant health across the country.

## Introduction

Giving birth in a healthcare facility with the guidance of skilled healthcare providers allows access to necessary medical interventions, ensures timely postnatal care, facilitates early detection of potential health issues, and ultimately leads to better health outcomes for both the mother and the child (Lee et al., 2022).

In contrast, a low level of institutional delivery has contributed to the underutilization of organized healthcare resources, resulting in a high maternal mortality rate, with 810 daily maternal deaths recorded globally in 2017 (World Health Organization, 2019a). An overwhelming proportion (94%) of these deaths occurred in low- and middle-income countries (LMIC), with a significant contribution from Sub-Saharan Africa (SSA) (World Health Organization, 2014, 2019a, 2019b). Furthermore, the absence of medical attention during pregnancy and childbirth can result in severe and long-lasting health issues (World Health Organization, 2014).

Various challenges and barriers impede institutional delivery, particularly in SSA, where less than 60% of births are attended by skilled personnel, in contrast to 96% in high-income countries (World Health Organization Regional Office for Africa, 2014). Among the East African countries, Ethiopia had the lowest institutional delivery rate, with a value of 26.2%, in stark contrast to the highest rate of 97% in Mozambique and a pooled rate of 87.47% for the entire region (Doctor, Nkhana-Salimu & Abdulsalam-Anibilowo, 2018; Central Statistical Agency (CSA) [Ethiopia] and ICF, 2016; Tesema & Tessema, 2021; Uganda Bureau of Statistics, 2017; Were et al., 2017).

The multifaceted dynamics that influence institutional delivery can be classified into demand- and supply side factors. Among supply side factors, distance to healthcare facilities significantly affects physical accessibility (Kumar, Dansereau & Murray, 2014; McGuire, Kreif & Smith, 2021; Nesbitt et al., 2016; Dotse-Gborgbortsi et al., 2020). Furthermore, travel time to healthcare facilities, influenced by factors such as transportation infrastructure quality, geographic landscape, and the economic conditions of mothers, also plays a pivotal role (Dahab & Sakellariou, 2020; Weiss et al., 2020). These interrelated factors intricately shape access to healthcare services, particularly childbirth (Dotse-Gborgbortsi et al., 2022; Karra, Fink & Canning, 2017; McGuire, Kreif & Smith, 2021). Moreover, this nexus between healthcare accessibility and the likelihood of institutional delivery is closely tied to urban-rural residence, contributing to disparities in healthcare access (Dotse-Gborgbortsi et al., 2022; Gao & Kelley, 2019; Dotse-Gborgbortsi et al., 2020).

Ethiopia has undertaken initiatives to improve institutional delivery and maternal health, including the expansion of the healthcare infrastructure (DeMaria, Smith & Berhane, 2022; Ethiopia Ministry of Health, 2010, 2015). This may have reduced the proximity to health facilities over time, coupled with interventions and policies to enhance institutional delivery (Berelie et al., 2020; Zewdu Amdie, Landers & Woo, 2022). The Ethiopian Demographic and Health Survey (EDHS) reported an increase in institutional delivery rates from 10% in 2011 to 26% in 2016 (Central Statistical Agency (CSA) [Ethiopia] and ICF, 2012, 2016). However, limited evidence exists on households’ distance from nearby health facilities and their impact on institutional delivery rates at the national scale. Thus, this study investigates the role of distance on institutional delivery trends over time by integrating data from two rounds of the EDHS survey with geographic positioning system (GPS) information for households and health facilities, as well as Ethiopian road network data.

## Methods

### Study setting

Ethiopia is one of the world’s ancient states and the second most populous country in Africa with a population of over 128 million (Worldometer, 2024). Ethiopia is a low-income country with a GDP *per capita* of 936 USD and low levels of income inequality (The World Bank, 2021). Although maternal delivery care is provided by the public, private, and non-governmental healthcare sectors, most of the care is provided by the public sector. The public health sector has a three-tier system comprising Primary Health Care Units (PHCUs), General Hospitals and Specialized Hospitals. The PHCU includes primary hospitals, health centers, and satellite Health Posts (Tiruneh, McLelland & Plummer, 2020).

### Data source

Data analysis was conducted by integrating data from two repeated cross-sectional EDHS (2011 and 2016) (Central Statistical Agency (CSA) [Ethiopia] and ICF, 2012, 2016), a spatial database of the geocoded Master Health Facility List for Africa (Maina et al., 2019), and Ethiopian road network data (World Bank Group, 2017). The sampling frames used for both the 2011 and 2016 EDHS were derived from the Population and Housing Census conducted in Ethiopia in 2007. The detailed sampling procedure for these surveys has been reported elsewhere (Central Statistical Agency (CSA) [Ethiopia] and ICF, 2012, 2016).

In this study, we included all women who participated in the 2011 and 2016 EDHS and had given birth within the 5 years preceding each survey. To ensure the accuracy and representativeness of the data, we employed complex sampling and weighting techniques in accordance with the guidelines provided by the DHS program (Elkasabi, 2015). Specific design variables were used for weighting purposes, including the individual weight of women (V005/1000000), primary sampling units (V021), and sample strata for sampling errors (V022). Incomplete data were excluded from the analysis. The analysis was conducted using weighted samples comprising 22,881 women residing in 14,951 households across 1,295 villages (see Table 1).

**Table 1:** Sample of respondents for most recent births in the 5 years preceding the 2011 and 2016 EDHS.

	Year of EDHS		Total
	2011	2016	
Women	11,858	11,023	22,881
Households	7,758	7,193	14,951
Villages/clusters	650	645	1.295

The spatial database of the geocoded Masters Health Facility List for Africa (Maina et al., 2019) provided information on the exact geographic locations of all public health facilities in the region in 2019. Therefore, it contains a list of all the public health facilities in Ethiopia and their GPS coordinates. Nonetheless, the GPS coordinates of the EDHS data were deliberately altered to safeguard the privacy of study participants. Urban clusters were shifted within a range of up to two kilometers (0–2 km) and rural clusters were shifted within a range of up to five kilometers (0–5 km). Furthermore, 1% of random rural clusters (every 100th cluster) are displaced up to 10 kilometers (0–10 km) (Burgert et al., 2013). Consequently, distance calculations were conducted using the adjusted cluster coordinates. Moreover, the Ethiopian road network dataset comprises vector digital data detailing primary and secondary road networks along with their traffic patterns and conditions across the country. This dataset was initially generated by the Ethiopian Road Authority and further refined by Africon Limited (World Bank Group, 2017).

The spatial database lists 5,215 healthcare facilities in Ethiopia. Among these facilities, 60% were primary health facilities (clinics) and 40% were hospitals.

### Variables

The dependent variable was institutional delivery. To facilitate the analysis, responses regarding the place of delivery were categorized as follows: if the birth took place at home, on the way to a health facility, or with a neighbor, it was coded as “home delivery”. Conversely, if the birth occurred at a hospital, primary healthcare center, health post, or sub-health post, it was coded as “institutional delivery”.

In addition, this study examined whether several variables are associated with institutional delivery. Specifically, it examined the relationship between the distance to the closest health facility and institutional delivery. Additionally, it explored the role of individual, household, and obstetric characteristics of the mothers, including their age at delivery, current marital status, employment status, educational status, religion, ethnicity, household wealth index, residence, region, husband’s education, husband’s work status, antenatal visits, and birth order.

### Statistical analysis

We used ArcGIS Desktop 10.8.2, to compute the road network distance between each EDHS cluster and the nearest health facility. The GPS coordinates of households and health facilities were integrated with the country’s road network vector digital data to create a network layer that defines road paths and connectivity. Network analysis tools in ArcGIS were used to compute the road network distance between each DHS cluster and the nearest health facility. Households within the same cluster were assigned the same distance value to reflect the common accessibility conditions.

In addition, using SPSS version 27, we calculated descriptive indices and conducted chi-square tests. Bivariate and multivariable logistic regression analyses were also conducted to identify potential factors that might influence institutional delivery. Variables that demonstrated statistical significance in bivariate regression were subsequently included in the multivariate regression analysis.

## Result

### Background characteristics of study participants

The respondents’ characteristics are listed in Table 2. In both surveys, most of the women lived in rural areas. In the 2011 EDHS, a significant majority (62.8%) of mothers who did not give birth at health facilities did not receive antenatal care (ANC), whereas in the 2016 EDHS, this percentage was slightly lower (49.8%). In both survey rounds, more than 66% of the women had no education and approximately 2% had attained higher education. In both 2011 and 2016, less than 5% of the women were covered by health insurance. Frequencies and percentages were weighted according to the guidelines outlined in the DHS program (Elkasabi, 2015).

**Table 2:** Background characteristics of women with the most recent birth in the 5 years preceding the 2011 and 2016 EDHS surveys.

Variables	EDHS 2011 (*N* = 11,858)			EDHS 2016 (*N* = 11,023)		
	Institutional delivery (freq. %)		*P*-value	Institutional delivery (freq. %)		*P*-value
	Yes	No		Yes	No	
Place of residence						
Urban	761 (64.5)	766 (7.2)	0.00	963 (33.3)	252 (3.1)	0.00
Rural	419 (35.5)	9,912 (92.8)		1,929 (66.7)	7,878 (96.9)	
ANC attendance						
No antenatal visits	135 (14.5)	4,379 (62.8)		237 (9.8)	2,581 (49.8)	
1	31 (3.3)	321 (4.6)	0.00	72 (3.0)	263 (5.1)	0.00
2	45 (4.8)	471 (6.8)		177 (7.3)	431 (8.3)	
3	192 (20.7)	790 (11.3)		555 (23.0)	845 (16.3)	
4 and above	518 (55.8)	990 (14.2)		1,360 (56.5)	1,055 (20.4)	
Don’t know	7 (0.8)	20 (0.3)		8 (0.3)	7 (0.1)	
Mothers age category						
<20	45 (3.8)	446 (4.2)	0.00	146 (5.1)	232 (2.9)	0.00
20–34	960 (81.4)	7,534 (70.6)		2,182 (75.5)	5,728 (70.4)	
35–49	175 (14.8)	2,698 (25.3)		563 (19.5)	2,171 (26.5)	
Education level						
No education	390 (33.0)	7,828 (73.3)	0.00	1,159 (40.1)	6,125 (75.3)	0.00
Primary	479 (40.6)	2,728 (25.6)		1,085 (37.5)	1,866 (23.0)	
Secondary	185 (15.7)	80 (0.7)		398 (13.8)	116 (1.4)	
Higher	127 (10.8)	41 (0.4)		251 (8.7)	23 (0.3)	
Religion						
Orthodox	695 (58.8)	3,818 (35.8)	0.00	1,379 (47.7)	2,393 (29.4)	
Protestant	196 (16.6)	2,560 (24.0)		564 (19.5)	1,765 (21.7)	0.00
Muslim	270 (22.8)	3,941 (36.9)		915 (31.6)	3,646 (44.8)	
Others	21 (1.8)	351 (3.3)		34 (1.2)	327 (4.0)	
Occupation						
Did not work	536 (45.8)	4,953 (46.8)		1,451 (50.2)	4,676 (57.5)	
Sales	233 (19.9)	1,701 (16.1)	0.00	497 (17.2)	799 (9.8)	0.00
Agriculture	114 (9.8)	3,124 (29.5)		469 (16.2)	1,990 (24.5)	
Skilled manual	121 (10.4)	687 (6.5)		133 (4.6)	272 (3.3)	
Others	166 (14.2)	124 (1.2)		342 (11.8)	394 (4.8)	
Wealth index						
Poorest	55 (4.7)	2,652 (24.8)		279 (9.6)	2,357 (29.0)	
Poorer	86 (7.3)	2,565 (24.0)	0,00	470 (16.3)	2,050 (25.2)	0.00
Middle	76 (6.4)	2,359 (22.1)		507 (17.5)	1,773 (21.8)	
Richer	156 (13.2)	2,114 (19.8)		546 (18.9)	1,453 (17.9)	
Richest	807 (68.4)	987 (9.2)		1,090 (37.7)	498 (6.1)	
Health insurance coverage						
No	1,143 (97.0)	10,645 (99.7)	0.00	2,718 (94.0)	7,915 (97.3)	0.00
Yes	35 (3.0)	29 (0.3)		174 (6.0)	216 (2.7)	
Ethnicity						
Oromo	358 (30.3)	4,090 (38.3)		858 (29.6)	3,739 (46.0)	
Amhara	474 (40.1)	2,501 (23.4)	0.00	791 (27.3)	1,580 (19.4)	0.00
Tigrie	94 (8.0)	643 (6.0)		429 (14.8)	304 (3.7)	
Somalie	24 (2.0)	341 (3.2)		88 (3.0)	397 (4.9)	
Others	231 (19.6)	3,104 (29.0)		963 (28.7)	2,107 (25.9)	
Husband’s education level						
No education	248 (21.5)	5,619 (52.9)		836 (31.0)	4,167 (53.7)	0.00
Primary	489 (42.4)	4,376 (41.2)	0.00	991 (36.7)	3,124 (40.3)	
Secondary	204 (17.7)	379 (3.6)		477 (17.7)	321 (4.1)	
Higher	207 (17.9)	169 (1.6)		381 (14.1)	90 (1.2)	
Don’t know	6 (0.5)	75 (0.7)		16 (0.6)	58 (0.7)	
Husband’s/partners occupation (grouped)						
Professional/technical/managerial	154 (13.4)	199 (1.9)	0.00	245 (9.1)	172 (2.2)	
Sales	262 (22.8)	657 (6.2)		308 (11.4)	403 (5.2)	0.00
Agriculture	343 (29.8)	9,014 (85.3)		1,252 (46.3)	5,635 (72.6)	
Skilled manual	231 (20.1)	390 (3.7)		282 (10.4)	283 (3.6)	
Others	161 (14.0)	286 (2.9)		615 (22.8)	1,269 (16.3)	
Current marital status						
Married	985 (83.4)	9,383 (87.9)	0.03	2,661 (92.0)	7,678 (94.4)	
Living with partner	71 (6.0)	540 (5.1)		41 (1.4)	83 (1.0)	0.03
Divorced	53 (4.5)	359 (3.4)		89 (3.1)	174 (2.1)	
Others	72 (6.1)	395 (3.7)		102 (3.5)	196 (2.4)	
Mother working currently						
Yes	525 (44.5)	2,839 (28.3)	0.00	1,023 (35.4)	1,965 (24.2)	0.00
No	655 (55.5)	7,208 (71.7)		1,869 (64.6)	6,165 (75.8)	
Birth order						
1	474 (40.1)	1,780 (16.7)		995 (34.4)	1,064 (13.1)	
2–3	412 (34.9)	3,282 (30.7)	0.00	975 (33.7)	2,384 (29.3)	0.00
4–5	165 (14.0)	2,563 (24.0)		478 (16.5)	2,126 (26.1)	
6+	130 (11.0)	3,053 (28.6)		444 (15.4)	2,557 (31.4)	
Sex of household head						
Female	298 (25.2)	1,467 (13.7)	0.00	507 (17.5)	1,022 (12.6)	0.00
Male	883 (74.8)	9,211 (86.3)		2,385 (82.5)	7,109 (87.4)	

### Trends of change in distance to health facilities

**Figure 1:**
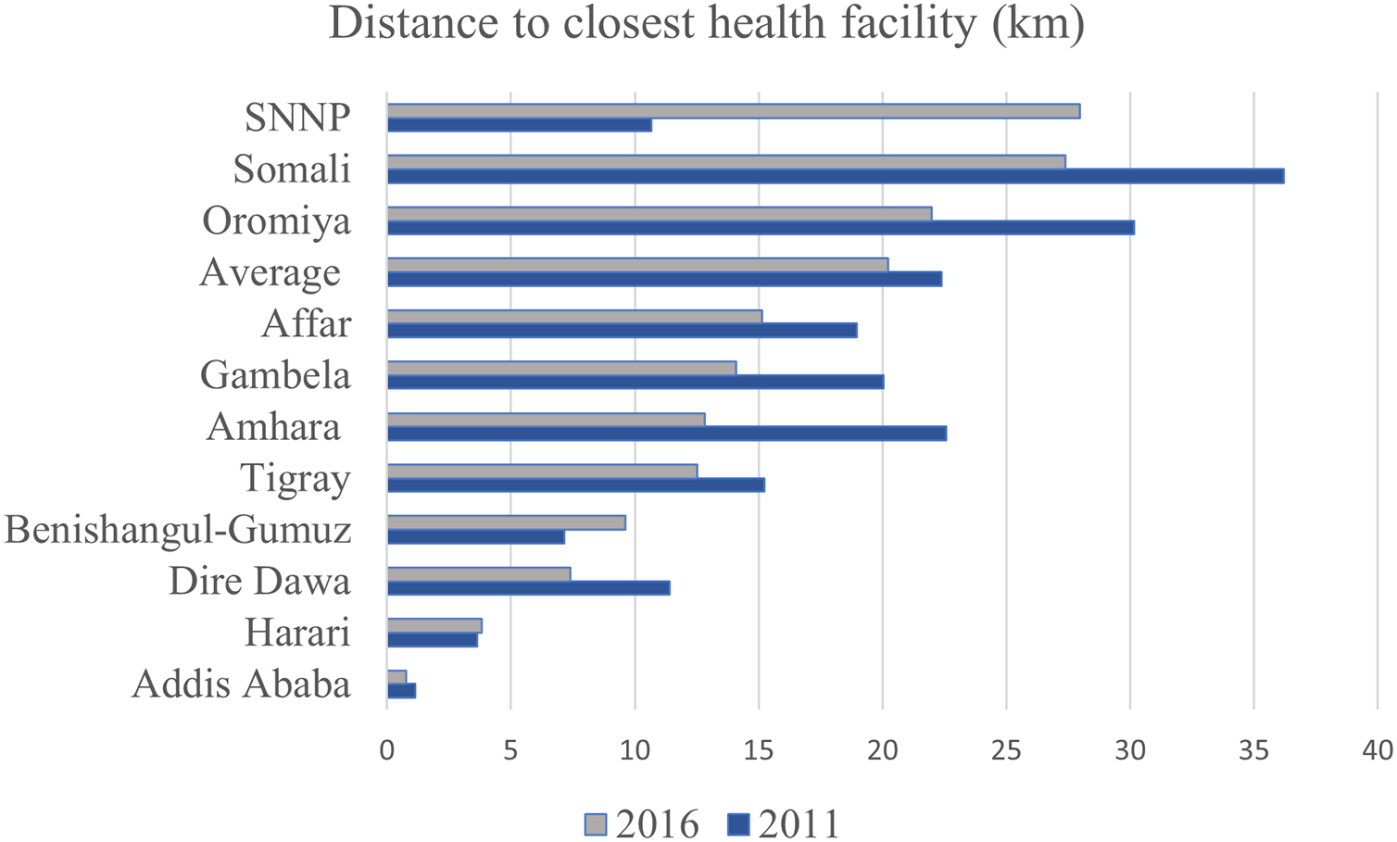
Mean distance of health facilities across different regions of the country for most recent births in the 5 years preceding the 2011 and 2016 EDHS.

The mean distance (in kilometers) of households to their closest public health facilities varies across Ethiopia (Fig. 1). The national average transportation distance was 22.4 km (95% CI [17.3–27.4]) in 2011 and 20.2 km (95% CI [15.9–24.6]) in 2016. Except for three regions (Southern Nations, Nationalities, and Peoples Region (SNNPR), Benshangul-Gumuz, and Harari), the mean distance between villages and facilities decreased between 2011 and 2016 (see Fig. 1).

**Figure 2:**
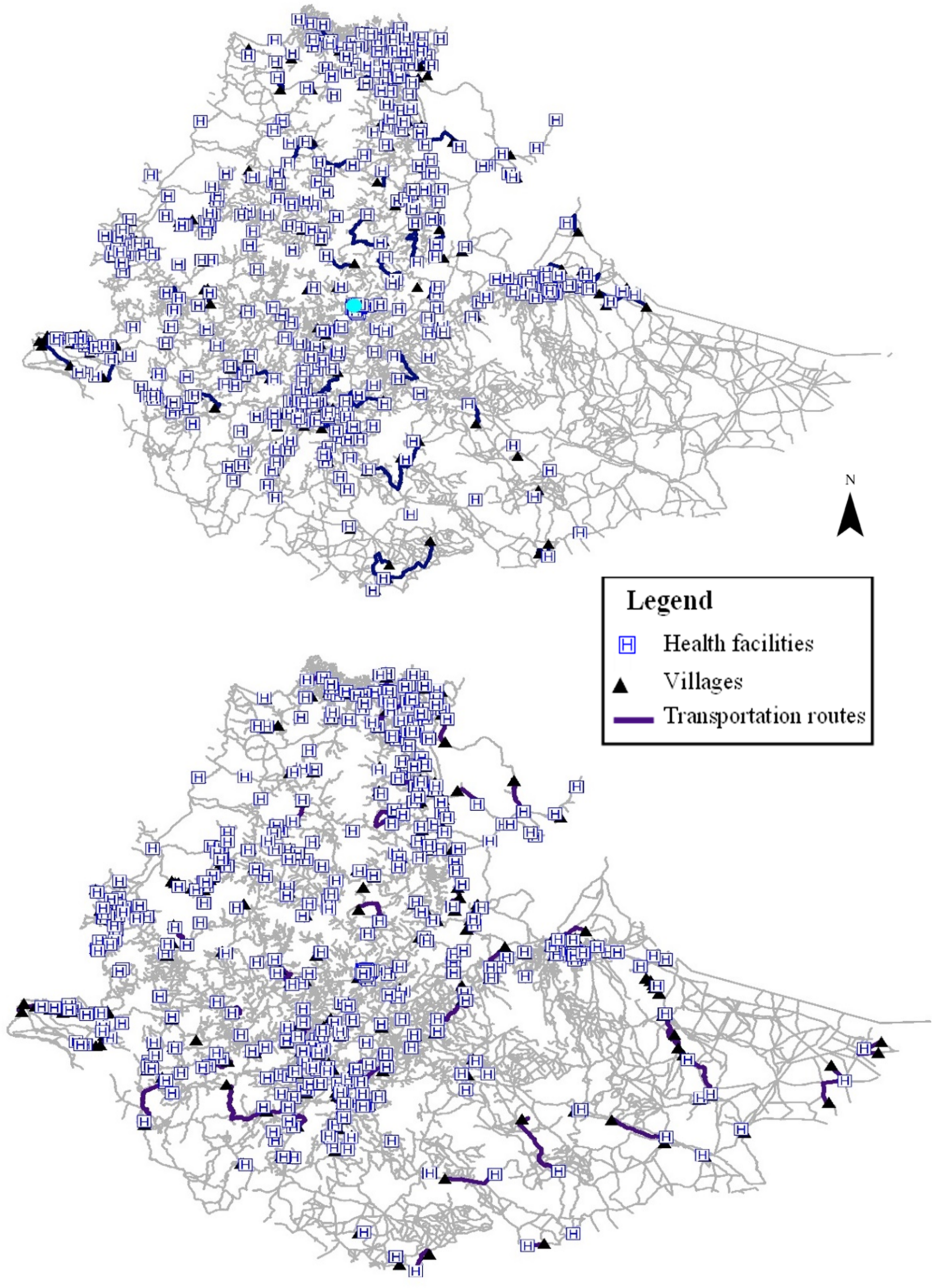
Geographical distribution of health facilities and shortest transportation routes from households of mothers delivered in 2011 (Top) and 2016 (Bottom) EDHS rounds. Map produced using data from Demographic and Health Surveys (DHS) (https://www.dhsprogram.com/data/available-datasets.cfm), Ethiopian road network (https://data.humdata.org/dataset/roads-network?) and the geocoded Master Health Facility List for Africa (https://data.humdata.org/dataset/health-facilities-in-sub-saharan-africa). Map source: ArcGIS.

The geographical distribution of health facilities and their shortest transportation routes from the villages of mothers who delivered in the two rounds of EDHS are shown in Fig. 2.

On the left side of the figure, the map shows the locations of the health facilities available in the 2011 EDHS along with the shortest transportation routes from the villages of the mothers who delivered during that period. Similarly, the map on the right-hand side displays the distribution of health facilities in the 2016 round and the corresponding shortest transportation route.

### Trends of institutional delivery

**Figure 3:**
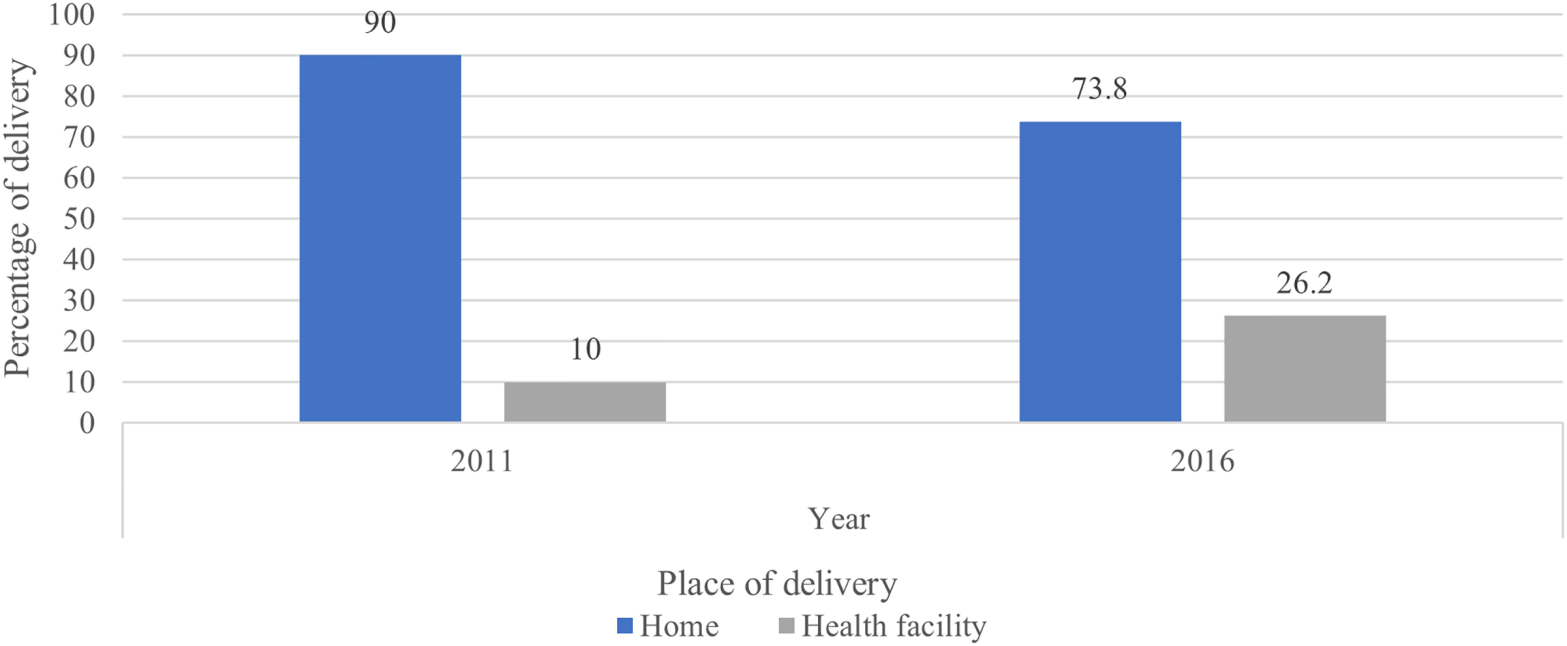
Place of delivery for most recent births in the 5 years preceding the 2011 and 2016 EDHS.

**Figure 4:**
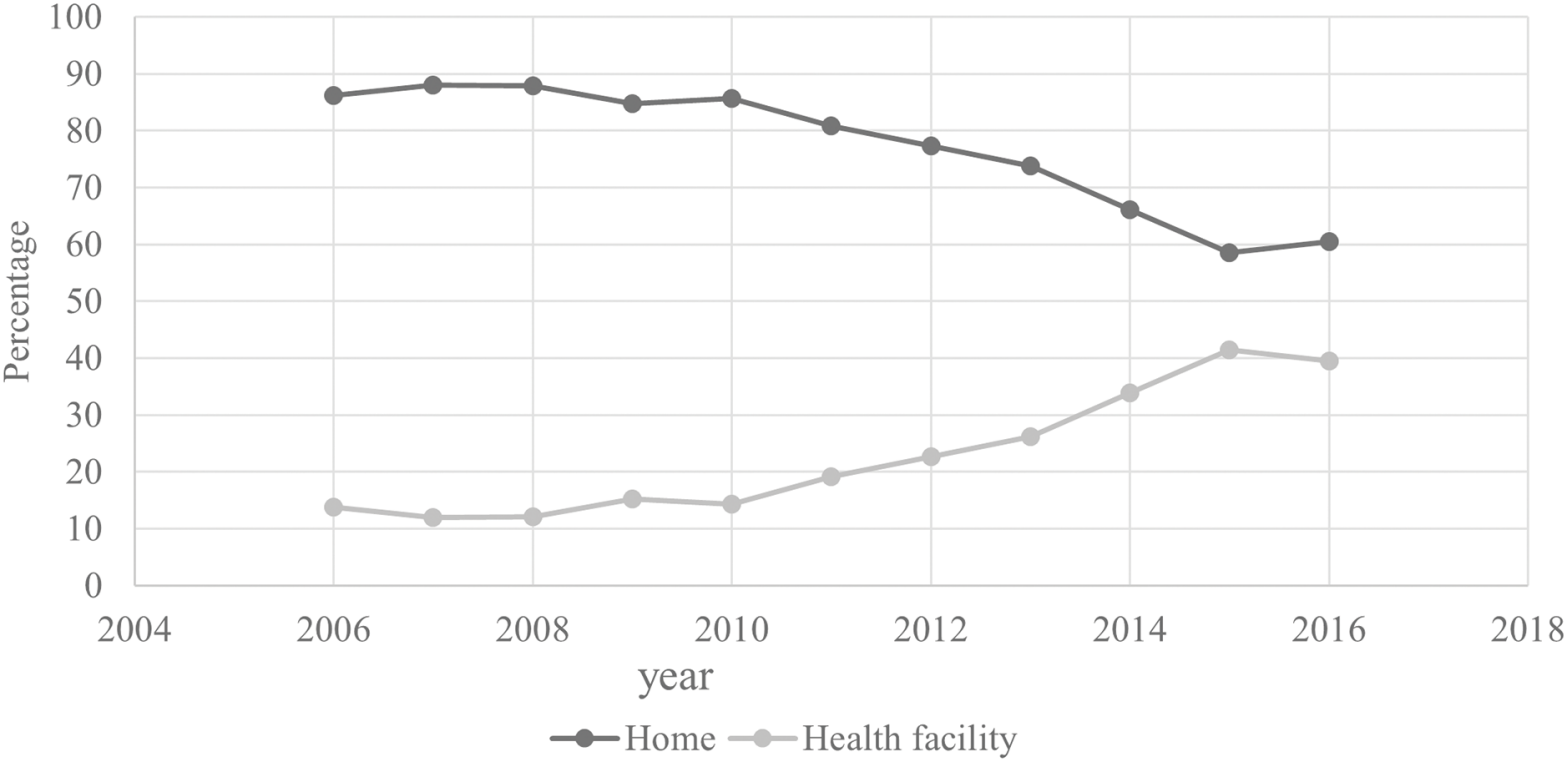
Trends of place of delivery for most recent births in the cumulative 10 years preceding the 2011 and 2016 EDHS.

Figure 3 shows the changes in institutional delivery rates across survey rounds. In 2011, 90% of births occurred at home compared to 73.8% in 2016. Figure 4 shows the rate of institutional delivery over time for the most recent births, between 2006 and 2016. As shown in the figure, the proportion of births in health facilities increased over the study period from 13.8% in 2006 to 22.7% in 2012, and 39.5% in 2016.

### Change in the determinants of institutional delivery in 2011 and 2016

Table 3 shows the factors associated with institutional deliveries in Ethiopia between 2011 and 2016. All models were estimated using a logistic regression analysis. For both surveys, Model 1 solely considered the distance between the woman’s village and the nearest health facility (measured in kilometers), whereas Model 2 encompassed all independent variables. As shown in Model 1, the farther a woman lived from the nearest healthcare facility, the less likely she was to choose healthcare facilities for delivery. A one-kilometer increase in the distance to the nearest health facility reduced the likelihood of delivering at a health facility by 1% (OR = 0.99, 95% CI [0.98–0.99], *p* < 0.05) in the EDHS 2016. Distance to health facilities tended to remain an important driver when other variables were controlled for in EDHS 2011.

**Table 3:** Determinants of institution delivery in Ethiopia in 2011 and 2016.

	EDHS 2011		EDHS 2016	
Variables	(1)	(2)	(1)	(2)
	Odds ratio	Odds ratio	Odds ratio	Odds ratio
	(95% CI)	(95% CI)	(95% CI)	(95% CI)
Distance to nearest health facility (Km)	0.99 [0.98–1.00]	0.99 [0.99–1.00]*	0. 99 [0.98–0.99]**	0.99 [0.99–1.00]
Number of ANC visit (compared with No ANC visit)				
Unknown		5.68 [1.19–27.22]**		3.29 [1.03–10.49]**
1		4.73 [1.00–22.34]**		2.02 [1.26–3.26]***
2		7.83 [1.67–36.78]***		3.67 [2.31–5.82]***
3		11.98 [2.64–54.33]***		4.92 [3.50–6.89]***
4 and above		2.52 [0.56–11.39]		7.35 [5.28–10.23]***
Age of the mother (compared with 35–49 age group)				
<20		0.63 [0.27–1.46]		0.88 [0.49–1.57]
20–34		1.08 [0.69–1.67]		0.88 [0.65–1.20]
Education level (compared with no education)				
Primary		1.81 [1.23–2.66]***		1.25 [1.01–1.55]**
Secondary		3.65 [1.69–7.84]***		2.28 [1.38–3.76]***
Higher		3.65 [1.42–9.31]***		3.13 [1.38–7.14]***
Current marital status (compared with married)				
Living with partner		0.55 [0.26–1.12]*		1.36 [0.95–1.0]
Divorced		0.91 [0.53–1.55]		–
Others		0.74 [0.38–1.43]		–
Mother working		1.41 [0.85–2.33]		1.35 [0.95–1.92]*
Occupation (compared with not working)				
Sales		0.66 [0.35–1.23]		0.79 [0.59–1.05]
Agriculture		0.76 [0.44–1.34]		1.03 [0.63–1.67]
Skilled manual		1.49 [0.69–3.24]		0.87 [0.49–1.55]
Others		1.36 [0.52–3.57]		0.66 [0.45–0.98]**
Ethnicity (compared with Oromo)				
Amhara		1.32 [0.83–2.08]		1.19 [0.80–1.709]
Tigrie		0.57 [0.30–1.06]*		4.58 [2.77–7.57]***
Somalie		1.52 [0.69–3.32]		1.23 [0.59–2.58]
Others		0.68 [0.42–1.07]*		1.18 [0.78–1.78]
Birth order number (compared with 6 and above)				
one		2.36 [1.30–4.29]***		2.60 [1.73–3.90]***
2–3		1.22 [0.73–2.07]		1.45 [1.06–1.98]**
4–5		0.79 [0.49–1.31]		1.15 [0.84–1.58]
Religion (compared with Muslim)				
Orthodox		1.35 [0.89–2.04]		0.89 [0.62–1.30]
Protestant		1.13 [0.61–2.09]		0.59 [0.37–0.95]**
Others		1.43 [0.64–3.22]		0.35 [0.14–0.90]**
Wealth index (compare to richest)				
Poorest		0.43 [0.23–0.81]**		0.30 [0.19–0.48]***
Poorer		0.45 [0.24–0.86]**		0.57 [0.38–0.86]***
Middle		0.41 [0.21–0.82]**		0.60 [0.40–0.90]**
Richer		0.48 [0.28–0.81]***		0.66 [0.46–0.94]**
Husbands/partners occupation (compare to agriculture)				
Professional/technical/managerial		0.81 [0.32–2.02]		0.77 [0.45–1.32]
Sales		1.56 [0.97–2.52]*		1.19 [0.79–1.77]
Skilled manual		1.38 [0.88–2.11]		0.88 [0.60–1.27]
Others		1.29 [0.76–2.21]		1.12 [0.83–1.50]
Husbands/partners education level (compare to no education)				
Primary		1.07 [0.77–1.52]		1.04 [0.81–1.34]
Secondary		1.40 [0.79–2.49]		2.00 [1.25–3.23]***
Higher		1.89 [0.75–4.73]		3.13 [1.59–6.15]***
Don’t know		0.41 [0.46–3.57]		0.72 [0.35–1.47]
Urban residence		5.01 [3.06–8.37]***		4.19 [2.21–7.96]***
Female household head		1.37 [0.93–2.04]		1.16 [0.84–1.61]

**Notes:**

*** *p* < 0.01.

** *p* < 0.05.

* *p* < 0.1.

In both surveys, it was observed that women who attended more ANC visits, had higher levels of education, had a lower birth order, possessed greater wealth, and lived in urban areas were more inclined to give birth in healthcare facilities. These factors were consistent despite slight variations in their impact over time. For instance, in the EDHS 2011 and 2016, the odds of women in the poorest socioeconomic group delivering at healthcare facilities were 0.43, 95% CI [ 0.23–0.81], *p* < 0.05) and 0.30, (95% CI [0.19–0.48], *p* < 0.01) times lower than those in the richest group, respectively. Similarly, mothers who had three ANC visits were about twelve (OR = 11.98, 95% CI [2.64–54.33], *p* < 0.01) and five (OR = 4.92, 95% CI [3.50–6.89], *p* < 0.01) times more likely to deliver at facilities than those who had no ANC visits in the EDHS 2011 and 2016, respectively. In the 2016 survey, women living in urban areas were approximately five times more likely to give birth in healthcare facilities in 2011 (OR = 5.01, 95% CI [3.06–8.37], *p* < 0.01) and four times (OR = 4.19, 95% CI [ 2.21–7.96], *p* < 0.01), respectively.

Overall, the results for potential determents showed a similar pattern in 2016 and 2011, with a few noteworthy differences related to age group, birth order, husband/partner education, sex of the household head, religion, and ethnicity. Notably, in 2016, more educated husbands/partners influenced their partners to give birth at healthcare facilities. For instance, mothers with a husband/partner with a higher educational level were three times more likely to deliver at health facilities (OR = 3.13, 95% CI [1.59–6.15], *p* < 0.01) than those with an uneducated husband/partner. On the other hand, the likelihood of Tigrayan women delivering to health facilities was higher in 2016 than in 2011 (OR = 4.58, 95% CI [2.77–7.57], *p* < 0.01) *vs* OR = 0.57, 95% CI [0.30–1.06], *p* < 0.1). Similarly, Protestant mothers were more likely to deliver at healthcare facilities in 2011 than they were in 2016.

## Discussion

This study examined the role of health facility distance on institutional delivery in Ethiopia. Despite the notable increase in the rate of institutional deliveries, rising from 10% to 26.2% between 2011 and 2016, and a slight reduction in the average distance to healthcare facilities by 2.2 km during this period, the likelihood of women giving birth at healthcare facilities remained low. Notably, while the impact of distance to healthcare facilities was modest, it tended to play a significant role throughout the timeframe.

This study underscores the considerable hurdles women face in accessing timely and safe maternal healthcare. Specifically, it reveals that women in labor may need to travel more than 20 km on average to reach a healthcare facility *via* the road network, highlighting the formidable barriers to accessing essential maternal health services. These challenges are further compounded by factors such as poor road infrastructure (Abdulkadr et al., 2022), limited transportation accessibility (Mushir & Hailemariam, 2015), financial constraints that inhibit transportation payments, and limited capacity of healthcare systems, which are common in many low-resource settings, including Ethiopia.

Furthermore, the role of distance in institutional delivery seems far from a straightforward one-dimensional relationship (Chen et al., 2022). For instance, the distance to healthcare facilities can impact the cost of accessing care due to transportation expenses and affect a woman’s ability to easily reach the facility. This is particularly critical in settings such as Ethiopia, where road network coverage is less than 41% of that required in the country (WFP, 2022) and limited-capacity healthcare systems pose significant challenges (Shiferaw et al., 2015; Yitbarek et al., 2023). It is evident in the current study where regions with a greater average distance to healthcare facilities, such as Somalia and Afar had lowest institutional delivery, which is consistent with previous studies (Awol, Edosa & Jemal, 2023; Karra, Fink & Canning, 2017; McGuire, Kreif & Smith, 2021). This underscores the pivotal role of the distance from healthcare facilities as gatekeepers in terms of accessibility (Chen et al., 2022). However, women living closer to healthcare facilities are more likely to complete ANC visits, which could motivate them to opt for institutional delivery, resulting in a virtuous cycle of delivery at health facilities (Dotse-Gborgbortsi et al., 2022).

Regression analysis also indicated that mothers who were more educated, had a lower birth order, completed more ANC visits, lived in wealthier urban households, and cohabited with more educated husbands were more likely to deliver at health facilities. These determinants remained consistent across both rounds of the survey despite the overall increase in institutional delivery. This is in line with previous research from 74 LMICs countries (Hasan et al., 2021) and Ethiopia (Fikre & Demissie, 2012; Gabrysch & Campbell, 2009; Gedilu, Debalkie & Setegn, 2018), which found that more educated women were more likely to deliver in healthcare facilities (Tesema & Tessema, 2021). This can be explained by multiple prospective pathways. For instance, more educated women are likely to have greater access to financial resources, greater health knowledge, and increased awareness of the potential benefits of institutional delivery (Mitikie, Wassie & Beyene, 2020). Additionally, they are more likely to cohabit with more educated partners, which can positively influence social norms and household decision-making. In line with previous studies (Gedilu, Debalkie & Setegn, 2018; Mitikie, Wassie & Beyene, 2020; Yoseph et al., 2020), our analysis also showed that women who completed more ANC visits were more likely to deliver at facilities, possibly because ANC visits build relationships with healthcare providers and foster trust (Hailemariam et al., 2023).

In terms of household characteristics, our study, as well as previous studies (Gabrysch & Campbell, 2009; Hasan et al., 2021; Yoseph et al., 2020), found that women living in wealthier households were more likely to deliver in facilities. A possible explanation could be that wealth is correlated with education or that women from wealthier backgrounds find it easier to cover the costs of healthcare and travel. Husbands’ education was another important factor that was significantly and positively associated with institutional delivery in the 2016 EDHS, as found in other studies (Mitikie, Wassie & Beyene, 2020; Yoseph et al., 2020). One possible reason for this is that educated husbands are more likely to have occupations that cover health costs and related fees. Simultaneously, they may be more receptive toward modern medicine, informed of the advantages of institutional delivery, and better able to communicate with health care professionals.

This study had three main limitations. First, by calculating the distance to the nearest facility, we implicitly assumed that the women wanted to visit their closest health facility. This could introduce bias if women perceive quality to be poor in these facilities or if levels of absenteeism are high and facilities are *de facto* closed. Second, we did not examine cultural factors, although they have been identified as being important in previous studies. Finally, we did not investigate the quality of care in health facilities, which was not possible due to the lack of data.

Nonetheless, the findings of this study are relevant to policymakers for several reasons, as it is the first nationally representative evidence of the role of distance from Ethiopia, and the first study to use data on the individual location of each village, facility, and road network in the country. Although we have identified other determinants of institutional delivery, this finding highlights the need to increase the number of healthcare facilities, particularly in rural regions. In addition, given that travelling long distances can be cumbersome for women when infrastructure is poor, infrastructure quality needs to be improved, particularly in rural areas (Dotse-Gborgbortsi et al., 2022). Although building more healthcare facilities is achievable in the short term, staffing is likely to be a key limitation. Ethiopia suffers from a severe shortage of healthcare staff, as there are only 0.96 healthcare workers per 1,000 people, which is far below the WHO target of 4.45 per 1,000 population (Haileamlak, 2018; World Health Organization, 2016). Hence, an increase in the number of health facilities is likely to increase the recruitment and training of health care providers. Second, we highlight the importance of the factors that are likely to take longer to change. Whether women deliver at health facilities or not is associated with their broader socioeconomic status (education and wealth) and husbands. This underlines the fact that low levels of institutional delivery in Ethiopia are likely to have deep-rooted causes. Hence, the goal of improving institutional delivery is likely to go hand-in-hand with other improvements in people’s lives, which are likely to be achievable only in the long term.

In terms of future research, given the modest impact of distance from healthcare facilities on institutional delivery rates in Ethiopia, and considering the average national transportation distance of over 20 km and the road network coverage of less than 41%, further research is recommended to explore the effects of infrastructure improvement, specifically road network and reduction in distance, which might influence institutional delivery. Regional studies could provide insights into local variations and needs, whereas longitudinal research may reveal the long-term effects of infrastructure improvement on institutional delivery.

## Conclusion

The impact of distance from healthcare facilities on institutional delivery in Ethiopia, although modest, remains notable. Over time, the rate of institutional delivery increased from 10% to 26.2%, in parallel with a reduction in the average national health facility distance from 22.4 to 20.2 km between 2011 and 2016. Despite this progress, institutional delivery rates remain lower than those in many other sub-Saharan African countries. Key factors influencing institutional delivery, such as education, antenatal care, socioeconomic status, urban residence, and partner education, have remained consistent across both surveys, underscoring the importance of addressing both physical accessibility and socioeconomic conditions. To improve maternal and infant health, policymakers and researchers should adopt a comprehensive approach that incorporates these determinants into policy development.

## Supplemental Information

10.7717/peerj.18128/supp-1 Supplemental Information 1 Ethiopia roads network.

10.7717/peerj.18128/supp-2 Supplemental Information 2 Codes used during analysis.

10.7717/peerj.18128/supp-3 Supplemental Information 3 Geocoded Master Health Facility List for Africa.

## Acronyms

ANC: Antenatal Care
DHS: Demographic and Health Surveys
EDHS: Ethiopian Demographic and Health Survey
GDP: Gross domestic product
GIS: Geographic information system
GPS: Global Positioning System
LMICS: Low and Middle-Income Countries
PHCUs: Primary Health Care Units
SSA: Sub-Saharan Africa
USD: United States dollar
WHO: World Health Organization

## Additional Information and Declarations

### Data Availability

The following information was supplied regarding data availability: The data and code utilized in our analysis and the Ethiopian road network data and a geocoded Master Health Facility List for Africa are available in the Supplemental Files. While we are unable to share the DHS data directly due to contractual restrictions (it was available for peer review), we have encoded the categories of certain DHS variables for transparency and replicability purposes. Interested parties can obtain access to the original DHS data from the DHS program website (https://www.dhsprogram.com/data/available-datasets.cfm) by fulfilling the specified requirements. For inquiries or comments regarding access to The DHS Program data, please contact the DHS administrator overseeing access requests at archive@dhsprogram.com.
