# Supplementary material for: The nexus between geographical distance and institutional delivery trends in Ethiopia: evidence from nationwide surveys
Source: PeerJ. 2024 Sep 20;12:e18128. doi: 10.7717/peerj.18128 (PMC11418825; doi:10.7717/peerj.18128)
Supplement: Supplemental Information 2 [file peerj-12-18128-s002.docx]

**Codes used during analysis.**

| Variable name in our analysis  (Variable label in DHS data) | Original values in DHS | Coded as: |
| --- | --- | --- |
| Mothers age category  (Age in 5-year groups) | 15 -19  20 - 24, 25-29, 30 -34  35 – 39, 40 -44, 45 -49 | <20  20 -34  35 -49 |
| Religion  (Religion) | Orthodox  Protestant  Muslim  (Catholic, traditional and others) | Orthodox  Protestant  Muslim  Others |
| Occupation  (respondents’ occupation – grouped) | Did not work  Sales  Agricultural – employe  Skilled manual  (Professional/technical/managerial,  Clerical, agricultural self-employee, household and domestic, services, unskilled manual, others, don’t know) | Did not work  Sales  Agriculture  Skilled manual  Others |
| Ethnicity  (Ethnicity) | Oromo  Amhara  Tigrie  Somalie  (Affar, Agew-Awi, Agew Hamyra, Alaba, Anyiwak, Argoba, Ari, Arborie, Bacha, Basketo, Bench, Berta, Bodi, Brayle, Burji, Bena, Chara, Dasenech, Dawuro, Debase/Gewada, Derashe, Dimi, Dizi, Donga, Dime, Fedashe, Gamo, Gebato, Gedeo, Gedicho, Gidole, Goffa, Gumuz, Guragie, Guagu, Hadiya, Harari, Hamer, Irob, Kefficho, Kembata, Konta, Komo, Konso, Kore, Koyego, Kunama, Karo, Kusumie, Malie, Mao, Mareko, Mashola, Mere, Me'enite, Messengo, Mejenger,, Mossiye, Mursi, Murle, Nao, Nuwer, Nyangatom, Oida, Qebena, Qechem, Qewama, Shekecho, Sheko, Shinasha, Silte, Sidama, Surma, She, Timebaro, Tsemay, Upo, Welaita, Werji, Yem, Zeyese, Zelmam, Other Ethiopian Ethinic Group, From different Parents, Eritrean, Djiboutian, Somalian, Kenyan, Sudanese, Other Foreigners) | Oromo  Amhara  Tigrie  Somalie  Others |
| Husband’s/partners occupation  (Husband/partner's occupation (grouped)) | Professional/technical/managerial  Sales  Agricultural – self-employed.  Skilled manual  (Did not work, clerical, agricultural- employee, household and domestic, services, unskilled manual, don’t know) | Professional/technical/managerial  Sales  Agriculture  Skilled manual  Others |
| Current marital status  ( | Married  Living with partner  Divorced  (never in union, widowed, no longer living together/separated) | Married  Living with partner  Divorced  Others |
| Birth order  (birth order number) | 1 – 17 | 1  2-3  4-5  6+ |
| Place of delivery | (Public sector, government hospital, government health center, government health station/clinics, government health post, other public sector, privet sector, private hospital, private clinic, NGO health facility, other private sector)  (Home, respondent’s home, other home, other) | Institutional  Not institutional |
